# Does neurodevelopmental load predict cognitive flexibility?

**DOI:** 10.1016/j.scog.2026.100446

**Published:** 2026-05-15

**Authors:** Alexandre Carpentier, Alexandre Durand, Mickael Naassila, Laurent Lecardeur, Marie-Cécile Bralet

**Affiliations:** a Service Pathologies Résistantes (SPR), Pôle Hospitalo-Universitaire Évaluation et Parcours de Soins en Psychiatrie Adulte, Centre Hospitalier Isarien-EPSM de l'Oise, Clermont de l'Oise, 60600, France; b Université Picardie Jules Verne, Amiens, 80000, France; c Institut de Psychiatrie (CNRS GDR 3557), Paris, 75000, France; d Service CRISALID-HDF, centre support de réhabilitation psychosociale et de remédiation cognitive, Évaluation et Parcours de Soins en Psychiatrie Adulte, Centre Hospitalier Isarien – EPSM de l'Oise, Clermont de l'Oise, 60600, France; e INSERM UMR 1247-Groupe de Recherche sur l'Alcool et les Pharmacodépendances (GRAP), Université de Picardie Jules Verne, Centre Universitaire de Recherche en Santé, Amiens, 80000, France; f DueL, 08 Quai des Docks, 06300, Nice, France

**Keywords:** Cognitive flexibility, Schizophrenia, Bipolar disorder, Neurodevelopmental disorder, Level of education

## Abstract

**Introduction:**

Cognitive flexibility (CF) impairments are a hallmark of schizophrenia (SCZ), bipolar disorder (BD) and neurodevelopmental disorders (NDD). These impairments are associated with functional outcomes and are influenced by neurodevelopmental and neurodegenerative processes.

**Methods:**

A retrospective analysis of 135 clinical records (75 SCZ, 29 BD, 31 NDD) was conducted. CF impairments were assessed using the Trail Making Test part B (TMT-B), with impairments defined as scores ≤ −2 standard deviations (SD). Multiple linear regression was used to adjust for confounding factors.

**Results:**

Rates of CF impairments were significantly higher in NDD (64.5%) and SCZ (57.3%) groups compared to the BD group (34.5%). No significant difference was observed between NDD and SCZ groups. After adjusting for confounders, the mean education level emerged as the only significant factor influencing CF impairments.

**Discussion:**

Our findings suggest that CF impairments are transdiagnostic and influenced by the level of education. This highlights the importance of considering environmental and sociodemographic factors in cognitive outcomes and supports the interplay between neurodevelopmental and neurodegenerative models, where education would appear to mitigate the impact of early neurodevelopmental abnormalities and delaying cognitive decline.

**Conclusion:**

CF impairments are shaped by shared vulnerabilities across diagnostic categories, with the level education playing a critical role. Future research should explore longitudinal trajectories and neurobiological mechanisms underlying CF impairments to inform targeted interventions.

## Highlights

• The level of education influences cognitive flexibility across diagnostic categories.
• Neurodevelopmental burden may be mitigated by level of education.
• Cognitive decline may be reduced by high levels of education.
• Results underline the interplay between two psychopathological models.

## 1 Introduction

Psychiatric and neurodevelopmental disorders are associated with cognitive impairments. For instance, approximately 80% of patients with schizophrenia (SCZ) and 30% of those with bipolar disorder (BD) had cognitive impairments (Harvey et al., 2022; Solé et al., 2017). In patients with neurodevelopmental disorders (NDD), the presence of cognitive impairment is a key feature criterion (Association, 2013; Thapar et al., 2017). These cognitive impairments are particularly correlated with functional outcomes, especially executive functions impairments, including inhibition, working memory, and cognitive flexibility (CF)(Huang et al., 2020; Mason et al., 2018; Mora et al., 2013; Rabinovici et al., 2015). CF refers to the ability to switch from one task or goal to another either spontaneously or in response to external stimuli (Hohl and Dolcos, 2024). CF is commonly assessed with neuropsychological tasks that require switching between rules, response sets, or mental operations, such as the Wisconsin Card Sorting Test, set-switching paradigms, Stroop-type interference tasks, verbal fluency switching tasks, and trail-making procedures (Hohl and Dolcos, 2024). Among these instruments, the Trail Making Test Part B (TMT—B) is one of the most widely used measures in clinical and research settings because it provides a brief, standardized, and easily administered assessment of executive control during alternating sequencing (Bowie and Harvey, 2006). In contrast to Part A, which mainly requires sequential number connection, TMT-B requires the participant to alternate between numbers and letters in ascending order, thereby placing specific demands on set-shifting and cognitive flexibility (Bowie and Harvey, 2006). Some authors indicates that TMT-B performance is related to perseverative behavior on the Wisconsin Card Sorting Test, further supporting its relevance as a measure of cognitive flexibility (Kortte et al., 2002). In addition, methodological studies have shown that TMT-B combines clinical usefulness, strong standardization, and broad applicability across neurological and psychiatric populations, which makes it especially suitable for comparative studies in routine practice (Bowie and Harvey, 2006). Because cognitive flexibility contributes to adaptive goal-directed behavior across developmental and clinical contexts, its assessment is particularly relevant when comparing young adults. Indeed, it has been shown to promote success in academic and professional settings as well as a successful transition to adulthood (Burt and Paysnick, 2012; Diamond and Lee, 2011). Similarly, efficient CF may mitigate the effects of cognitive aging, enhance resilience to stress, and improve quality of life (Burke et al., 2019; Davis et al., 2010; Genet and Siemer, 2011). The efficient development of CF depends on the proper maturation of brain structures, particularly the prefrontal cortex (Dajani and Uddin, 2015; Tong et al., 2024). CF impairments are recognized as a key feature across psychiatric and NDD. NDD, which include disorders like autism spectrum disorder (ASD) and attention-deficit/hyperactivity disorder (ADHD), have an early onset, often before school age (Association, 2022). This early impact on brain development would present a higher neurodevelopmental burden than in SCZ or BD, where the onset is typically later. The long-standing impairments in NDD could disrupt cognitive pathways during critical stages of neurodevelopment, potentially leading to persistent and more severe CF impairments (Uddin, 2021). In contrast, SCZ and BD typically emerge in late adolescence or early adulthood, and the evolution of cognitive impairments in these disorders follows a more gradual trajectory (Emre Bora, 2015a; Bortolato et al., 2015). In patients with SCZ, cognitive impairments, including CF impairments, often precede the clinical features but stabilize at the time of diagnosis (Fusar-Poli et al., 2012). According to several authors, cognitive impairments would stabilize or worsen proportionally to the early onset of neurodevelopmental abnormalities (Bora, 2015b; Nuechterlein et al., 2014; Sheffield et al., 2018). In patients with BD, cognitive impairments are reported at least until diagnosis of first psychosis episode, to be less severe than in patients with SCZ, particularly regarding CF, which may reflect less significant neurodevelopmental abnormalities (Salagre et al., 2020). Both neuroprogression and/or neurodegenerative processes have been suggested to explain the cognitive decline and accelerated brain aging in BD. However, several authors suggest that the cognitive impairments observed in some patients with BD may follow a similar trajectory to the cognitive impairments found in patients with SCZ (Bora, 2015a; Bortolato et al., 2015). These patients may also have early neurodevelopmental abnormalities and genetic risk factors shared with patients with SCZ. To our knowledge, only two studies have compared these three populations (Ardesch et al., 2023; Kushima et al., 2022). These studies focused on differences in genetic and neuroimaging factors. Moreover, to our knowledge, no study has compared cognitive impairments, particularly CF impairments, across the young patients of these three populations. Nevertheless, a comparative study of CF among these three populations early in life would provide further insight into the early pathophysiology underlying these disorders. The primary objective of this work was to compare CF abilities among a group of patients with SCZ, with BD, and with unspecified NDD. We hypothesized that patients with unspecified NDD would have higher rates of CF impairments due to the early onset of the disorders and neurodevelopmental abnormalities. The secondary objectives were to explore the variables associated with CF impairments across the three groups and to identify any significant similarities or differences between them.

## 2 Methods

Our study was a retrospective analysis of 135 patient clinical records (75 SCZ, 29 TB, 31 NDD). Data were prospectively collected from a psychosocial rehabilitation care department (CRISALID-HDF) and a specialized department for adult's patients with NDD (SITED) in the Public Mental Health Institution of Clermont de l'Oise from 2018 to 2024. The existence of a CF impairment was defined by a Trail Making Test part B (TMT—B) score expressed as standard deviation (SD) ≤ −2. The severity of CF impairments using SD as a quantitative variable was also used.

## 3 Results

Approximately one-third of the patients in each group were women. The mean age at the time of cognitive assessments was 30.9 ± 7.93 years in the SCZ group, 43.6 ± 13.0 years in the BD group, and 27.0 ± 7.39 years in the NDD group. The mean level of education was comparable between SCZ and BD groups with 15.0 ± 2.69 years and 16.0 ± 3.40 years respectively. The mean level of education in the NDD group was significantly lower (12.5 ± 3.42 years), compared to SCZ group (H = 27.5, *p* = 0.001) and BD group (H = 39.3, *p* < 0.0001). The mean duration of illness was significantly lower in SCZ group (11.8 ± 9.04 years) compared to the BD group (24.3 ± 14.9 years) and NDD group (18.3 ± 8.47 years). Our results showed significant CF impairments in patients with NDD and patients with SCZ compared to patients with BD (64.5% vs. 34.5%, χ^2^ = 5.41, *p* = 0.02, and 57.3% vs. 34.5%, χ^2^ = 4.37, *p* = 0.037, respectively). No significant difference in the presence of CF impairments was observed between NDD and SCZ groups (χ^2^ = 0.469, *p* = 0.493). The severity of impairments was highest in NDD group, followed SCZ group and BD group but without any statistically significant difference between the groups. However, after adjusting for confounding factors in a multiple linear regression, the mean level of education emerged as the only significant variable explaining the differences in CF impairments between the groups (B = −0.402, *p* = 0.002). Indeed, the higher the mean level of education, the lower rates of CF impairment. Moreover, there was a significant negative correlation between the severity of CF impairments and the mean level of education in the overall sample (r_s_ = − 0,311, *p* < 0.001). Neither age, gender, age of onset, duration of illness, the use of benzodiazepines or antipsychotics as well the dosage in diazepam or chlorpromazine equivalents, or a history of substance use significantly contributed to the observed differences in CF impairments between the three groups.

## 4 Discussion

Initially, our results indicated that patients with NDD and SCZ exhibited significantly higher rates of CF impairments compared to those with BD. Beyond these initial group differences, it is important to clarify how TMT-B performance should be interpreted in terms of CF. In a clinical sample, Kortte et al. showed that only a flexibility index from the Wisconsin Card Sorting Test (percentage of perseverative errors) significantly predicted TMT-B performance after accounting for age and TMT-A, whereas a measure of failure to maintain set was not associated with TMT-B (Kortte et al., 2002). These results support the view that TMT-B is particularly sensitive to cognitive flexibility, even though its performance also reflects more basic processes already captured by TMT-A (Bowie and Harvey, 2006). In this framework, our use of TMT-B as a primary index of CF appears consistent with previous work while acknowledging that it does not provide a process-pure measure of executive functioning (Bowie and Harvey, 2006). Our study was not designed to provide a comprehensive assessment of executive functioning, and we therefore focused on TMT-B as a pragmatic index of CF that is widely used and easy to implement in routine clinical practice. More broadly, our findings indicate that TMT-B performance should not be interpreted as a direct and isolated marker of diagnosis-specific CF impairments, but rather as the outcome of interactions between executive control and more general cognitive resources. Existing literature has documented higher rates of CF impairments in patients with SCZ compared to patients with BD (Salagre et al., 2020) and no difference between patients with NDD (ASD specifically) and patients with SCZ (Eack et al., 2013; Morais et al., 2024). To our knowledge, only one study has explored CF abilities between pediatric patients with NDD (ADHD specifically) and patients with BD with higher rates of CF impairments in patients of BD compared to patients with ADHD (Walshaw et al., 2010). However, a critical juncture in our analysis occurred with the adjustment of our statistical model to account for the level of education. After adjustment, the significant differences in CF impairments among the three groups were no longer observed. Indeed, logistic regression analysis revealed that a higher level of education was associated with a lower proportion of CF impairments, irrespective of diagnostic categories. This finding highlights that cognitive impairments may be influenced by shared factors, such as education irrespective of diagnosis. On one hand, this pattern is in line with methodological work on the Trail Making Test showing that performance on both Part A and Part B is strongly influenced by age, education and global intellectual abilities, and that normative interpretation should therefore rely on stratified or regression-based norms (Bowie and Harvey, 2006). Bowie and Harvey specifically emphasized the need to take education and IQ into account when interpreting TMT-B scores, because these variables substantially affect completion times and the likelihood of being classified as impaired. In this context, our finding that educational attainment fully accounted for the initially observed group differences in TMT-B performance suggests that part of what appears as diagnostic variation in CF may actually reflect differences in educational background and cognitive reserve. Moreover, the link between higher education and better TMT-B scores aligns with research showing education's benefits on executive functions (Lövdén et al., 2020). Formal education, accumulated over years, may foster the development of cognitive strategies and neural plasticity that support CF abilities (Hertzog et al., 2008). On the other hand, level of education has been theorized to contribute to cognitive reserve, which refers to the brain's ability to maintain cognitive function despite apparition of neurodegenerative disorder (Chapko et al., 2018). Studies have suggested that higher level of education would be associated with a reduced risk of dementia and a slower rate of cognitive decline (Du et al., 2023) notably in patients with BD (Matsumoto and Hamatani, 2024). The significantly lower mean level of education observed in our group of patients with NDD compared to the other groups might partially explain their initially lower TMT-B scores. NDD can sometimes affect educational attainment due to various factors such as learning difficulties, social challenges, and attentional issues (Nadeau et al., 2020). This interplay between neurodevelopmental trajectories and educational opportunities is crucial when considering the neurodevelopmental model. It is plausible that early neurodevelopmental differences impact both cognitive development directly and indirectly through their influence on educational engagement and attainment, ultimately affecting performance on cognitive tasks like the TMT-B (McCormick et al., 2020; Souissi et al., 2022). Taken together, these data suggest that TMT-B performance reflects not only core cognitive flexibility processes, but also the broader efficiency of working strategies and the availability of cognitive resources shaped by education and intellectual functioning. This view is consistent with studies of spatial working memory in schizophrenia showing that patients can display different strategic profiles despite comparable error rates, suggesting that executive and visuospatial components of working memory may be differentially taxed across individuals (Lecardeur et al., 2010).In practice, it is therefore important to consider both educational attainment and broader indices of intellectual functioning, even though these two concepts are not equivalent and their correlation is not necessarily positive. IQ describes a construct that encompasses several associated cognitive functions and essentially reflects a form of cognitive efficiency, which may interact with educational opportunities to shape performance on tasks such as TMT—B. Accordingly, group differences in TMT-B should be interpreted cautiously if they are not adjusted for education and, whenever possible, for IQ or validated proxies of intellectual level. Considering the neurodegenerative model in patients with SCZ and BD, where progressive cognitive decline can occur in some patients, our findings regarding education offer an interesting perspective (Buoli et al., 2017). Indeed, we did not find a significant correlation between the duration of illness and the intensity of CF impairments, but we did find a significant negative correlation between the intensity of CF impairments and the mean level of education regardless of the diagnostic category. Although the young age of our sample and the cross-sectional nature of our study prevents the assessment of neurodegenerative trajectories in SCZ or BD, the strong link between education and CF suggests that higher cognitive reserve from education may provide resilience against cognitive decline.(Barnett et al., 2006; Cotrena et al., 2021; Matsumoto and Hamatani, 2024).

Therefore, our study has several implications. Firstly, it reinforces the growing recognition of the importance of environmental and sociodemographic factors in shaping cognitive trajectories in both typical and atypical development. As McCutcheon et al. suggest (McCutcheon et al., 2025), transdiagnostic factors would represent a meaningful share of the variance in cognitive functioning in psychosis. Their study also found that lower levels of education were associated with lower cognitive scores across various diagnostic categories. Our findings support a dimensional approach to psychopathology, viewing cognitive impairments as transdiagnostic rather than tied to specific diagnostic categories (Kelly et al., 2018; Walther and Morrens, 2020). The significant influence of education on CF across diagnostic groups highlights the limitations of relying solely on categorical diagnoses to explain cognitive variability. Thirdly, our findings, suggest a potential link between the neurodevelopmental and neurodegenerative models. Education, as a proxy for cognitive reserve, would mitigate the impact of early neurodevelopmental abnormalities by promoting neuroplasticity and strengthening cognitive networks (Lövdén et al., 2020; Vance et al., 2010). This aligns with the neurodegenerative model, which emphasizes the progressive decline of cognitive functions due to structural and functional brain changes, such as synaptic loss and fronto-striatal circuit dysfunction in psychiatric disorders (Buoli et al., 2017; Stone et al., 2022). In this context, higher educational levels may act as a protective factor, delaying or reducing the severity of cognitive decline, particularly in patients with SCZ or BD (Buoli et al., 2017). Finally, to our knowledge, our study was the first study who compared CF abilities across young patients with NDD, SCZ and BD and focused on a geographically isolated cohort, which allowed us to control for environmental factors and potential stressors that may influence CF abilities. Indeed, several authors have shown significant variability in cognitive functions based on geographic location, both in the general population and in patients with psychiatric disorders.(Guzman et al., 2024; Lin and Chen, 2024; Steinberg et al., 2023).

Despite these insights, our study presents several limitations. The retrospective design limited our ability to gather detailed developmental histories and longitudinally track CF abilities trajectory. The heterogeneity within the group of patients with NDD, encompassing various conditions with potentially distinct neurodevelopmental trajectories and cognitive profiles, is a significant limitation (Craig et al., 2016). Future research should aim for more homogeneous samples within subgroups of patients with NDD to better understand the specific impact on CF abilities. Finally, the use of the TMT-B to assess CF may limit the generalization of our results. Although the TMT-B is widely used, other aspects of CF, such as verbal or conceptual flexibility, are not assessed, which may provide a partial view of CF abilities (Kopp et al., 2021). Moreover, using the TMT-B SD to define CF impairment may lack precision. Some authors suggest subtracting the TMT-A SD from the TMT-B SD to account for processing speed impairments, as slower processing can reduce CF and falsely indicate CF impairment (MacPherson et al., 2017). In addition, future studies should combine TMT-B with formal estimates of intellectual functioning to improve the interpretation of between-group differences and to better disentangle executive dysfunction from more global cognitive influences. This consideration is particularly important in transdiagnostic samples, where education and cognitive reserve-related factors likely explain a substantial proportion of the variance in TMT-B performance. However, the choice of TMT-B had important practical advantages in the context of this retrospective clinical study. Bowie and Harvey highlighted that TMT-A and TMT-B are brief, highly standardized, and show excellent inter-rater and alternate-forms reliability, which makes them particularly well suited for routine clinical use and repeated assessment (Bowie and Harvey, 2006). Although both TMT and WCST are sensitive to executive dysfunction, TMT-B requires less extensive concept formation and strategy learning, which reduces long-term practice effects and facilitates its use across heterogeneous clinical populations. These pragmatic and psychometric features justify the choice of TMT-B as our main measure of CF, while the limitations of this approach are explicitly acknowledged below.

In conclusion, despite its limitations, this study highlights the complex relationship between diagnostic categories, CF, and the critical role of education. Our findings suggest that cognitive impairments are often transdiagnostic, reflecting shared vulnerabilities or protective factors across conditions. The strong impact of education on CF emphasizes the need to consider socioeconomic factors and access to quality education as key influences on cognitive development and resilience in psychiatric and neurodevelopmental disorders. Our results also indicate that TMT-B performance should be interpreted in light of these background cognitive resources, rather than as a pure marker of diagnosis-specific CF impairments. Accordingly, future studies using TMT-B to compare clinical groups should systematically adjust for education and, whenever possible, for IQ or validated proxies of intellectual functioning in order to obtain a more accurate picture of disorder-related executive impairments. Future research should focus on longitudinal studies to explore CF trajectories and the role of education over time. Additionally, investigating the neurobiological links between CF impairments, diagnosis, and education, using tools like neuroimaging, could deepen our understanding of the mechanisms underlying these processes.

## CRediT authorship contribution statement

**Alexandre Carpentier:** Writing – review & editing, Writing – original draft, Methodology, Formal analysis, Conceptualization. **Alexandre Durand:** Writing – original draft, Data curation. **Mickael Naassila:** Writing – review & editing, Supervision. **Laurent Lecardeur:** Writing – review & editing, Validation, Supervision, Methodology, Conceptualization. **Marie-Cécile Bralet:** Writing – review & editing, Validation, Supervision, Methodology, Conceptualization.

## Funding statement

This research did not receive any specific grant from funding agencies in the public, commercial, or not-for-profit sectors.

## Declaration of competing interest

Marie-Cecile Bralet and Laurent Lecardeur have received honoraria for lectures and non-financial support from Boehringer, Otsuka, and Lundbeck, outside the submitted work. All other authors declare that they have no conflicts of interest.

## Data Availability

The data that support the findings of this study are available from the corresponding author, Alexandre Carpentier, upon reasonable request.
